# Correlational Linkages Between Doctor Empathy and Doctor-Rated Patient Adherence: An Interaction Ritual Chains Perspective

**DOI:** 10.3390/healthcare14152378

**Published:** 2026-08-04

**Authors:** Shumin Mai, Lu Li

**Affiliations:** 1 The Institute of Social and Family Medicine, School of Medicine, Zhejiang University, Hangzhou 310058, China; shumin_mai@zju.edu.cn; 2 Zhejiang Shuren University, Hangzhou 310015, China

**Keywords:** empathy, treatment adherence, interaction ritual chains, chronic disease management, doctor–patient communication

## Abstract

**Highlights:**

**What are the main findings?**

- Doctor empathy has a direct correlation with doctor-rated patient adherence.
- The interaction ritual chains theory can be used to primarily explore how doctor empathy relates to doctor-rated patient adherence in healthcare settings.

**What are the implications of the main findings?**

- Empathy and communication skills may be relevant to doctor-rated adherence, although intervention studies are needed to determine whether improving these skills leads to better adherence.
- Future research should further expand the research scope, enroll diverse research subjects and refine measurement instruments to further examine the validity of the research hypotheses and theoretical model.

**Abstract:**

**Background:** Relevant studies have demonstrated that patient adherence is one of the most important factors influencing treatment effectiveness. Failure to adhere to medical prescriptions can significantly hinder the achievement of desired therapeutic outcomes. Doctor empathy is presumed to correlate with patient adherence. However, the underlying pathways are not well understood. This study explores the associations linking doctor empathy to doctor-rated patient adherence using interaction ritual chains (IR chains), which provide a framework for investigating this process. **Methods:** From April 2023 to July 2023, a cross-sectional survey was administered to doctors from primary-level clinics in Zhejiang Province, China. Structural equation modeling was used to test the research hypothesis. **Results:** A total of 459 valid data entries from doctors were included in the analysis. The analysis result indicated that hypothesis H1: doctor empathy is positively correlated with doctor-rated patient adherence was supported. The standardized regression coefficient was 0.17 (*p* = 0.009). Hypothesis H2: emotional energy is hypothesized to mediate the correlational linkage between doctor empathy and doctor-rated patient adherence (standardized regression coefficient = 0.02, *p* > 0.05). Hypotheses H3: doctor treatment behavior is hypothesized to mediate the correlational linkage between doctor empathy and doctor-rated patient adherence (standardized regression coefficient = 0.02, *p* > 0.05), and hypothesis H4: emotional energy and treatment behavior are hypothesized to mediate the correlational linkage between doctor empathy and doctor-rated patient adherence (standardized regression coefficient = 0.02, *p* > 0.05) were not supported. **Conclusions:** The IR chains theory can be used to primarily explore how doctor empathy relates to doctor-rated patient adherence in healthcare settings. Doctor empathy shows a positive correlation with doctor-rated patient adherence.

## 1. Introduction

According to the 2022 World Health Statistics report published by the World Health Organization (WHO), the four major primary chronic disease categories (cancer, cardiovascular disease, diabetes, and chronic respiratory disease) were responsible for 33.2 million deaths globally in 2019, representing a 28% increase from 2000 [1]. Additionally, the WHO Global Physical Activity Status Report 2022 predicts that between 2020 and 2030, there will be nearly 500 million new cases of chronic diseases worldwide, with hypertension accounting for 47% of these cases [2]. In China, the Sixth National Health Services Statistical Survey (2021) [3] reported that the prevalence of chronic diseases among the surveyed population aged 15 years and older (comprising 256,000 individuals) stood at 35.1% in 2018, with hypertension and diabetes being the two most prevalent conditions. In 2019, chronic diseases accounted for 88.5% of all deaths of Chinese residents, and the premature mortality rate due to four categories of major chronic diseases, including cardiovascular and cerebrovascular diseases, cancers, chronic respiratory diseases and diabetes, was 16.5% [4]. Although a wide range of effective therapeutic drugs and interventions for hypertension and diabetes have been developed globally, the above data reveal that the prevalence of both conditions continues to rise, and treatment outcomes fail to reach ideal levels. Some studies have shown that patient adherence is one of the most important factors influencing the treatment effect [5,6]. Chronic diseases are characterized by long disease courses, with little possibility of spontaneous recovery or complete cure [2]. Their prevention and treatment require a sustained, ongoing commitment, emphasizing the crucial role of patient adherence to medical treatment. Taking hypertension and diabetes as examples, in addition to drug treatment, lifestyle interventions covering diet and physical exercise are also imperative. When patients fail to adhere to treatment or self-manage without professional guidance, achieving the desired therapeutic effect becomes challenging. This may lead to complications, disease progression, and even mortality [6]. Existing studies have indicated that treatment adherence among patients with chronic diseases requires improvement. As demonstrated by data from the China Health and Nutrition Survey, in 2015, the awareness, treatment, and control rates of hypertension among adults were 48.08%, 40.51% and 14.65%, respectively [7]. The treatment and control rates were insufficient. The substantial gap between awareness and these rates suggests a need to improve patient adherence. Good adherence to medical advice is associated with stable blood pressure and blood glucose levels, which are crucial factors in ensuring quality of life, diagnostic and therapeutic outcomes, and the quality of care [6].

Patient adherence to treatment is influenced by many factors. Patients and doctors are the primary participants in the process of disease treatment. Doctors have medical authority, enabling them to provide professional knowledge and technical guidance for patients’ disease treatment. When patients seek medical consultations, doctors need to effectively communicate therapeutic strategies and guide them toward scientific treatment based on their specific conditions, which essentially translates to treatment adherence. To achieve this objective, effective doctor–patient interaction and communication are indispensable. Some studies revealed that patients with better doctor–patient communication had better disease control (such as blood pressure control) [8]. Doctors must leverage their authoritative influence to provide psychological guidance, cognitive direction, and humanistic care for patients. Empathy is a crucial component of doctor–patient communication. It serves as an effective approach for doctors to exert their professional influence on patients through psychological guidance, cognitive direction, and humanistic care. Unlike the authority derived from pharmacological treatment, empathy can be cultivated and enhanced through doctors’ efforts within a relatively short timeframe. It represents both an essential professional competency for medical practitioners and a fundamental expectation that patients hold regarding compassionate care and understanding during medical consultations [9,10]. In recent years, empathy has garnered substantial attention for its critical role in enhancing doctor–patient communication. Studies indicate that doctor empathy can improve treatment adherence, thereby promoting better health and favorable outcomes. It also contributes to the quality of medical care, including patients’ quality of life, satisfaction, and the doctor–patient relationship [5,9,10]. However, the specific processes or pathways underlying the relationship between doctor empathy and patient outcomes require further investigation and exploration.

In 2004, Randall Collins introduced Interaction Ritual Chains (IR chains) theory [11]. This theory posits that social activities involving capital and emotional exchange can be conceptualized as interaction rituals. The core elements of IR chains include mutual focus of attention, shared emotional states, and emotional energy. Unlike existing doctor–patient communication models (such as the Four Habits Model [12], Patient-Centered Clinical Strategies Method [13], SEGUE Framework [14], and 6S Extended Model [15]) which primarily focus on standardized behavioral processes of doctors as single participants while neglecting diverse and dynamic real-world contexts, the IR chains theory offers a more systematic and comprehensive framework. It incorporates ritual components, outcomes, and interaction processes within participants’ contexts. Furthermore, whereas most existing models devote limited attention to emotional communication and offer only brief explanations, IR chains theory explicitly addresses situational contexts and momentary encounters among emotionally engaged individuals. It emphasizes how emotions and consciousness are transmitted through encounter chains [11]. As “emotion” significantly influences human behavioral choices, it constitutes a crucial component of IR chains, thereby addressing the traditional sociological research gap in emotional aspects [11]. This study aims to explore the association between doctor empathy and patient adherence within the context of disease diagnosis and doctor–patient communication. Specifically, we investigate emotional communication and influencing factors between doctors and patients. The IR chains theory provides a theoretical framework for this investigation. Most existing research on IR chains is predominantly qualitative, with quantitative studies remaining limited. Furthermore, its application in the medical field remains scarce.

As two of the most prevalent chronic diseases [2], hypertension and diabetes are frequently addressed in chronic disease management policies and have garnered significant government attention. In recent years, Zhejiang Province in China has actively promoted pilot programs for managing these ‘two chronic diseases’ (a term officially used in government policy documents to refer to hypertension and diabetes) [16], with the aim of improving the overall health of residents. These policies have provided a practical basis for this study’s investigation into the association between doctor empathy and treatment adherence among patients with hypertension and/or diabetes.

This study is designed to investigate the association between doctor empathy and doctor-rated patient adherence within Zhejiang Province’s macro-policy framework for managing the two chronic diseases (hypertension and diabetes). Based on the background of disease treatment and doctor–patient communication, we propose a research model integrating the IR chains and empathy theory from the perspective of doctors as healthcare providers. We will then examine the relationship between empathy and patient adherence empirically. Relevant strategies will be developed to improve chronic disease management and doctor empathy and communication skills.

## 2. Literature Review and Hypotheses Proposed

Empathy is a vital component of doctor–patient communication. It enables doctors to influence patients by offering psychological support, cognitive guidance, and humanistic care. In medical practice, empathy means understanding, considering, and caring for patients. By empathizing with their patients and gaining their trust, doctors can establish a trusting doctor–patient care partnership to help overcome disease. Although studies have demonstrated that empathy is related to patient treatment adherence, the underlying processes and pathways remain unclear. The core components of IR chains are mutual focus, a shared emotional state, and emotional energy. These are related to emotional communication and provide a theoretical framework for our study. Hypotheses and a theoretical framework are developed based on IR chains under clinical treatment scenarios.

### 2.1. The Concept of Empathy

In 1873, Robert Vischer first conceptualized “Empathy” and used the German term “Einfühlung” to describe the observer’s experience when viewing and appreciating works of art [17]. Although ‘empathy’ has an early origin, there is no unified definition, and different scholars and research fields offer different interpretations. In medical research, Mohammadreza Hojat (2007) [18] proposed empathy within the context of patient care as a cognitive attribute rather than an emotional one (distinguishing it from “Sympathy”), referring to the understanding (rather than feeling) of patients’ experiences, concerns, and perspectives, along with the ability to communicate this understanding. It is also called clinical empathy. This definition emphasizes the healthcare provider’s viewpoint in defining empathy toward patients. Some studies have approached the assessment of doctor empathy from the patient’s perspective. For instance, Kim et al. [19] proposed that patient perception of doctor empathy refers to the patient’s sense of being understood and accepted by their doctors. In the preliminary phase of this study, based on the literature review and research team discussions, we adapt Professor Hojat’s definition of empathy in the context of patient care to define doctor empathy toward patients as: the doctor’s proactive effort to identify, understand, and appreciate the patient’s perspectives, views, feelings, emotions, experiences, or circumstances from the patient’s standpoint, and to communicate this understanding effectively, thereby providing better care and concern while maintaining objective judgment.

### 2.2. The Concept of Interaction Ritual Chains

Within the framework of Interaction Ritual Chains (IR chains) [11], rituals are seen as relatively standardized outcomes of various behavioral postures aimed at forming and maintaining specific social relationships. These rituals encompass certain procedural activities or routine verbal interactions in daily life, such as greetings and congratulations, which constitute polite and habitual verbal exchanges [11]. Interaction rituals are everyday programmed activities conducted by participants (interactors) through the exchange of capital and emotions. They can be understood as activities in social life involving the exchange of capital and emotions [11].

The main components of interaction ritual chains include: (1) the gathering of two or more individuals in the same space, where their co-presence influences one another; (2) boundaries against outsiders, enabling participants to distinguish in-group members; (3) a mutual focus of attention among participants, concentrating on a shared object or activity; and (4) the sharing of common emotional or affective experiences among participants (Figure S1 in Supplementary File). When these components are effectively integrated, culminating in a high degree of mutual focus and emotional sharing, four primary outcomes emerge: group solidarity and a sense of membership; individual emotional energy characterized by confidence, enthusiasm, strength and the initiative to take action, with a degree of long-term stability; symbols representing the group, such as icons, words or gestures, that make members feel connected to the collective; a sense of morality that upholds justice within the group and respects group symbols. This sense of morality guards against violations, with moral guilt and anger among members arising from breaches of group solidarity or symbols [11].

### 2.3. Research Hypotheses and Model

When applying the IR chains, scholars analyze, explore, and refine them according to different research scenarios and purposes. Some studies primarily utilize specific elements or core variables mentioned in the IR chains [20,21,22], while others employ all elements of the theory to analyze stakeholder behaviors in specific scenarios [23,24,25]. The uniqueness of healthcare services means that the elements of ‘bodily co-presence’, ‘barrier to outsiders’ and ‘mutual focus’ are natural constituents of the ritual chain of interaction. Patients generally choose hospitals and doctors due to physical or psychological illness or discomfort, initiating a process in which both doctors and patients are present for disease diagnosis, treatment, and communication [24]. Patients disclose their medical conditions and personal information to doctors, who are obligated to protect patient privacy by excluding individuals unrelated to the medical condition, thereby establishing barriers to outsiders [24]. To diagnose and treat diseases, patients visit hospitals, and doctors are required to fulfill their duties by doing their best to treat patients. Thus, the disease or the patient’s health condition becomes the mutual focus for both parties [24]. The concept of ‘collective effervescence’ mentioned in the IR chains can be seen as a shared emotional experience among participants, such as ‘sharing joy and sorrow’, which reflects the accumulation of emotions to a certain extent and has an element of transience or spontaneity. During treatment, doctors need to remain objective and make calm decisions. When a patient’s condition is not optimistic or unstable, doctors may feel tense or even distressed. Conversely, when the condition is optimistic and stable, doctors may feel calm and joyful. Doctors who empathize with patients and achieve reasonable emotional synchrony can help maintain objective and scientific judgment during treatment. However, deep and intense emotional synchrony can impair a doctor’s ability to make accurate judgements. Therefore, this study primarily focuses on exploring “shared mood”, i.e., “empathy”. When doctor–patient communication is harmonious and smooth, it helps both parties to deepen their understanding and awareness of the disease (through reinforcement and feedback between shared emotional states and mutual focus). Some outcome elements mentioned in interaction rituals, such as ‘group solidarity’, ‘symbols of social relationship’, and ‘standards of morality’, can be summarized and elucidated through ‘the quality of medical care’, which emerges and develops alongside good doctor–patient interaction and communication.

This study takes patient adherence (evaluated by the doctor) as the outcome variable. Combining the characteristics of medical services and research objectives, and drawing on the two core components of the IR chains: “shared mood” and “emotional energy”. Doctors are responsible for healing and saving lives, and are equipped with the professional expertise and technical proficiency to fulfil this mission. From this perspective, the research proposes a hypothesis regarding the association between doctor empathy and doctor-rated patient adherence and establishes a corresponding research model.

#### 2.3.1. Hypothesis of the Direct Association Between Doctor Empathy and Patient Adherence (Doctor-Rated)

Medical services are characterized by professionalism and technicality, with doctors holding the advantage of diagnostic knowledge and technical skills, thus giving them authority in medical treatment. The process of treating disease is inseparable from professional and scientific medical guidance, and the effectiveness of treatment methods relies heavily on patients adhering to medical advice. Doctors must communicate these methods to patients and convince them to accept and adopt the advice, which will benefit disease treatment. This process involves doctors’ communication skills, psychological induction, cognitive guidance, and humanistic care authority. Empathy plays a crucial role in realizing this authority and providing psychological induction and cognitive guidance.

Patients have higher expectations of medical services due to increased health awareness, hoping not only for a cure, but also for a positive medical experience [26]. Afflicted by illness, patients are often burdened with negative emotions such as depression, anxiety, and panic, and expect more understanding, care, and help from doctors [18,27]. For patients with long-term chronic diseases, especially the elderly in vulnerable situations such as living alone or without companionship or care, the needs they have for doctors extend beyond medical care to include emotional expression, communication, and support [28].

Doctors’ empathy involves recognizing and understanding patients’ feelings, which can be seen as shared emotional states in the IR chains. This empathetic engagement is associated with a heightened sense of responsibility and enthusiasm, and it corresponds to a practice that replaces cold technical skills with warm, caring treatment methods. By listening to and communicating with patients, doctors can understand their condition, explain the causes, symptoms, and treatment methods clearly and simply, and answer questions patiently, encouraging patients to understand and accept medical advice. This approach fosters better patient adherence to treatment and contributes to improved clinical outcomes [17,29,30,31,32,33]. Based on this, the following hypothesis is proposed:

**H1.**  *Doctor empathy is positively correlated with patient adherence (doctor-rated).*

#### 2.3.2. Hypothesis of the Mediation Role of Emotional Energy

Emotional energy acts as a core driving force in interaction rituals. Individuals generate and accumulate positive emotions through ritual interactions, which may trigger positive behavioral chain reactions in subsequent social encounters [11]. Anger, fear, joy, and sadness are the basic human emotions, and sustained long-term emotions can form emotional energy. Emotional energy ranges from high confidence and enthusiasm at one end, through a neutral everyday state, to depression and low initiative at the other [11]. As a ‘driving force’, emotional energy influences the level of interaction and behavioral activities of participants. When sadness or depression becomes a prolonged emotional state characterized by low emotional energy, this reduces participants’ level of activity. This leads to physical fatigue and withdrawal, as well as making social interactions passive, sluggish, and perfunctory [11]. One study in the consumption area based on IR chains revealed that brand emotions (such as brand satisfaction, identification, and happiness) can mediate the relationship between consumer interaction and purchasing behavior [21]. Emotional energy can also mediate the influence of brand community interaction rituals (i.e., interpersonal and self-interaction rituals) on fan loyalty. Another study revealed that community members acquire positive emotional energy through interaction rituals, generating emotional resonance with each other and with their focus, thereby strengthening loyalty [34].

IR chains help explain doctor–patient communication during treatment. Doctors recognize and empathize with patients’ negative emotions from illness, and they express caring concern. These behaviors are linked to a stronger sense of responsibility, motivation, and enthusiasm, as well as to an authoritative stance in psychological guidance and humanistic care. Doctors’ care and warm attitude are linked to patients’ trust, as well as to greater acceptance of and adherence to treatment. In other words, doctors’ empathy may be positively associated with patients’ adherence to treatment by increasing emotional energy in doctors. Based on this, the following hypothesis is proposed:

**H2.**  *Emotional energy is hypothesized to mediate the correlational linkage between doctor empathy and patient adherence (doctor-rated).*

#### 2.3.3. Hypothesis of the Mediation Role of Doctor Treatment Behavior

Relevant research has demonstrated that empathy is related to altruistic and prosocial behaviors. It establishes a universal connection between one’s own emotional experiences and the well-being of others, serving as a source of helping behaviors. The higher one’s empathy level, the more frequently they engage in prosocial and altruistic acts such as helping others and sharing resources [35,36]. This study focuses on doctors’ behavioral expressions of empathy toward patients. These include providing medical instructions, explaining diagnoses and treatments, offering reminders and emotional support, and patiently addressing concerns. These behaviors contribute to patients’ physical and mental recovery and are manifestations of altruism. Patients suffering from illnesses often experience low mood and anxiety, and they expect doctors to provide more care and support [18,27]. Hypertension and diabetes predominantly affect elderly patients, who generally have lower comprehension and acceptance of medical knowledge than younger patients. This necessitates doctors explaining medical information in more patient and accessible ways. When doctors empathize—recognizing and understanding patients’ circumstances and emotions—they are more likely to explain conditions and treatments clearly, patiently answering questions [27,32]. By clearly and repeatedly reinforcing key points regarding hypertension and diabetes management, doctors can effectively communicate accurate treatment methods and techniques. This is associated with better patients’ understanding and acceptance of medical advice, guiding them towards appropriate treatment pathways and preventing adverse outcomes resulting from misconceptions or incorrect behaviors [29]. Such actions can positively relate to improving patient adherence. In other words, doctor empathy may be associated with patient adherence through specific treatment behaviors. Based on this, the following hypothesis is proposed:

**H3.**  *Doctor treatment behavior is hypothesized to mediate the correlational linkage between doctor empathy and patient adherence (doctor-rated).*

#### 2.3.4. Hypothesis of the Chain Mediating Role of Emotional Energy and Doctor Treatment Behavior

Relevant studies have shown that active empathy toward patients can reduce medical staff’s occupational burnout and elevate their work enthusiasm and motivation [18,37]. Empathy also increases their sense of responsibility, thereby influencing caring and therapeutic behaviors—such as understanding patients’ situations, providing more attention, and patiently explaining conditions [38]. Work responsibility, motivation, and enthusiasm are core components of emotional energy in IR Chains, which act as a driving force to positively shape doctors’ clinical behaviors. That is, emotional energy exerts a positive relationship with doctors’ treatment behavior. Furthermore, doctors’ empathetic expressions, warm and friendly service attitudes, and explanations and care provided to patients during treatment can improve patients’ understanding and acceptance of treatment plans. Thereby, this may be related to patients’ following medical advice and cooperating actively. Based on the previous discussion of the direct association between doctor empathy and patient adherence, and the mediation roles of emotional energy and doctor treatment behavior within the framework of IR chains, the following hypothesis is proposed:

**H4.**  *Emotional energy and doctor treatment behavior are hypothesized to mediate the correlational linkage between doctor empathy and patient adherence (doctor-rated).*

**Figure 1:**
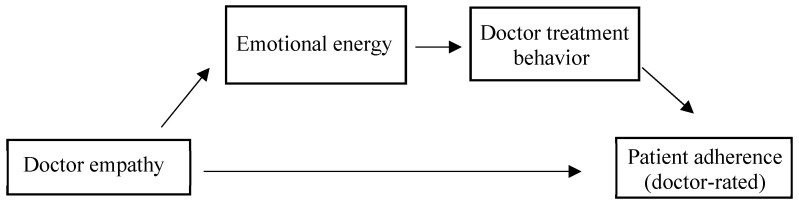
Research model.

The research model illustrating the association between doctor empathy and doctor-rated patient adherence from the doctor’s perspective is depicted in Figure 1.

## 3. Methods

### 3.1. Participants

In China, primary-level clinics play a significant role in providing healthcare services to residents, especially those in rural, economically deprived, or remote mountainous areas. In recent years, China has undergone a significant transformation in its healthcare system [39]. Multiple policies have been implemented to strengthen primary-care capacity, enabling the public to access quality medical services locally and reducing overcrowding in tertiary hospitals. For instance, integrated medical systems, telemedicine, and family doctor programs have been vigorously promoted nationwide. Many patients with chronic conditions (e.g., hypertension, diabetes, stroke) choose primary clinics for convenience, leading to frequent doctor–patient interactions. Accordingly, doctors’ attitudes and behaviors—including kindness, understanding, care, patience, and empathy—exert an impact on patient adherence and the quality of doctor–patient relationships. Therefore, this study targeted doctors employed at primary-level clinics as research subjects.

The survey included general practitioners, traditional Chinese medicine physicians, and rural doctors who provide daily outpatient diagnosis and treatment for patients with hypertension and/or diabetes (referred to as the “two chronic diseases”). The survey covered community health service centers and their subordinate stations, township health centers, and village clinics. The inclusion criteria were as follows: (1) holding a formal employment position at a medical institution; (2) possessing independent prescribing rights; (3) undertaking outpatient work and being responsible for managing patients with the two chronic diseases; (4) fluent Mandarin communication and clear self-expression; (5) voluntary participation in the questionnaire survey; and (6) signing written informed consent prior to participation.

### 3.2. Measurements

#### 3.2.1. Doctor Empathy

The Jefferson Scale of Physician Empathy for physicians and health professionals (JSPE-HP), developed by Prof. Hojat and his research team [40], has been widely applied and validated in studies measuring physician empathy. In 2009, Chinese scholars Professors Ma Li and Li Xiaohan [41] translated the scale into Chinese and evaluated its reliability and validity among nursing staff. For the current study, we revised the Chinese version of the scale to fit domestic linguistic habits and evaluate doctor empathy, after obtaining official authorization from Jefferson University (see Supplementary File for the authorization document) and the original translators, Professors Ma Li and Li Xiaohan. Our research team first completed the Chinese translation, then invited Professor Ma to review the translated items for semantic accuracy, readability, and alignment with clinical physician scenarios. Professor Ma also offered alternative translations for ambiguous items where necessary. The revised Chinese version was subsequently sent to bilingual researchers at Jefferson University for back-translation into English to evaluate cross-linguistic conceptual equivalence. All items were refined based on feedback, and Jefferson University officially approved the adapted scale as a valid tool.

The JSPE-HP contains 20 items across three dimensions: perspective-taking (recognizing and understanding patients’ viewpoints), compassionate care (understanding and responding to patients’ emotions and experiences) and standing in the patient’s shoes (adopting patients’ perspectives in decision-making). Items are rated on a 7-point Likert scale (1 = strongly disagree to 7 = strongly agree), with higher scores indicating stronger agreement. Ten items were reverse-scored during the analysis. Data with missing values exceeding 20% of all items (≥4 unanswered items) were eliminated, and missing values in the retained data were filled using the mean imputation method. (The reliability and validity of the scale can be checked in the Supplementary File: Table S7; the Cronbach’s alpha is 0.85).

#### 3.2.2. Doctor Emotional Energy

According to the relevant research [42,43,44,45], based on the results of interviews with doctors and discussions among the research team, a self-developed questionnaire was used to measure doctors’ emotional energy. The questionnaire consists of 11 items divided into three dimensions: six items measuring sense of responsibility, three items measuring work enthusiasm, and two items measuring sympathy for patients’ suffering. Responses are rated on a 5-point Likert scale (1 = strongly disagree, 5 = strongly agree), with higher scores indicating greater emotional energy. Questionnaires with missing data in more than 20% of items (≥3 blank items) were excluded; remaining missing values were processed via mean imputation. (The Supplementary File contains details of the development process of the doctor emotional energy questionnaire and its reliability and validity: Tables S1–S6 and S8; the Cronbach’s alpha is 0.92).

#### 3.2.3. Doctor Treatment Behavior

A self-developed questionnaire is used to measure doctor treatment behavior. The 11-item questionnaire mainly covers four aspects: delivering knowledge regarding the diagnosis and treatment of hypertension and diabetes, reminding patients to follow medical advice, providing emotional support for patients, and assisting patients in coping with disease-related difficulties. A 5-point Likert scale (1 = strongly disagree, 5 = strongly agree) was used to evaluate the items; higher scores indicate better treatment behavior. Meanwhile, data with more than 20% of items with missing responses (>3 items not answered by participants) were excluded, and any remaining missing values were addressed using mean imputation. (The development process of doctor treatment behavior and the reliability and validity of the questionnaire can be found in the Supplementary File: Tables S1–S6 and S9; the Cronbach’s alpha is 0.97).

#### 3.2.4. Doctor-Rated Patient Adherence

This study adopted a single-item indicator for doctor-rated patient adherence. Doctors were asked to evaluate the overall treatment compliance of their patients with hypertension and/or diabetes via the question: ‘How would you rate the overall treatment adherence of your patients with hypertension and/or diabetes?’ Doctors assess on a 5-point Likert scale with the following response options: ‘Very good’, ‘Good’, ‘Fair’, ‘Poor’, and ‘Very poor’, which are assigned scores of 5, 4, 3, 2, and 1, respectively. Responses of “unclear” or “unable to evaluate” are treated as missing values and subsequently imputed using the mean value. The survey targets grassroots general practitioners managing hypertension and diabetes patients long-term; frontline doctors have continuous and comprehensive observation of patients’ daily medication, diet, and follow-up behaviors, so their overall comprehensive evaluation has certain practical reference value in primary care settings.

#### 3.2.5. Control Variables

Doctors’ demographic characteristics, including gender, age, marital status and educational level, were included as control variables in the analysis to mitigate potential confounding effects on the results.

### 3.3. Data Collection

Cities in Zhejiang Province were classified into high, medium, and low economic tiers based on prefecture-level GDP rankings from the 2022 Zhejiang Statistical Yearbook [46]. High economic tier cities: Hangzhou, Ningbo; medium economic tier cities: Wenzhou, Shaoxing, Jiaxing, Taizhou, Jinhua, and Huzhou; low economic tier cities: Quzhou, Lishui, and Zhoushan. Due to dependence on local medical institutions for subject recruitment, convenience sampling was adopted. Considering data accessibility, field implementation feasibility, and institutional cooperation willingness, two cities were selected from each tier: Hangzhou and Ningbo (high economic tier); Taizhou and Jinhua (medium economic tier); and Quzhou and Lishui (low economic tier). A total of 100 questionnaires were planned to be collected from primary care doctors (including general practitioners, traditional Chinese medicine physicians, and rural doctors) in each selected city. Questionnaires were distributed via medical alliances, health bureaus, and family doctor association platforms, combining paper and electronic versions. Local institutional coordinators shared electronic questionnaire links through internal WeChat work groups or direct peer forwarding to target doctors. The sample size was determined based on recommendations suggesting it should be approximately 10–20 times the number of observed variables in the model [47]. With 43 observed variables, the final questionnaire required at least 430 (43 × 10) completed responses. A total of 504 questionnaires were initially collected from April to July 2023. A total of 504 questionnaires were collected. After the exclusion of invalid questionnaires, such as (1) doctors did not sign the informed consent form; (2) more than 20% of the items or questions in the questionnaire were left unanswered; or (3) over 80% of the variables/content assessed using Likert scales were rated at the same level; 459 valid responses remained, with the valid questionnaire rate of 91.07%.

### 3.4. Data Analysis

Data were cleaned, organized, and analyzed using Excel 2016 (Microsoft Corporation, Redmond, WA, USA) and SPSS 20.0 (IBM Corp., Armonk, NY, USA). Missing values were imputed with the mean. Structural equation modeling (SEM) was conducted using AMOS 20.0 (IBM Corp., Armonk, NY, USA) to test the research hypotheses and assess the overall model fit. SEM is a multivariate statistical analysis technique that combines aspects of factor analysis and multiple regression to analyze complex relationships among observed and latent variables. It can handle multiple dependent variables simultaneously and tests hypothesized causal models, typically represented as path diagrams [48].

To detect potential common method bias (CMB) originating from single-source self-reported doctor data, Harman’s single-factor test was performed on all measurement items of doctor empathy, emotional energy, doctor treatment behavior, and doctor-rated patient adherence via unrotated exploratory factor analysis. The results indicated that the first extracted common factor explained 35.95% of the total variance, below the critical threshold of 40% [49,50], which suggests that common method variance is relatively limited in its impact on the present sample. (The analysis result of CMB can be checked in the Supplementary).

## 4. Results

### 4.1. Demographic Characteristic

The formal questionnaire survey was carried out from April to July 2023. Considering data accessibility, the operability of field research arrangements, and institutional cooperation willingness, participants were physicians recruited from 21 community health service centers and 25 township health centers across multiple districts and counties of six cities in Zhejiang Province: Hangzhou (Yuhang, Shangcheng), Ningbo (Haishu, Yinzhou), Taizhou (Xianju), Jinhua (Yongkang), Lishui (Suichang), and Quzhou (Kaihua). Based on preliminary interviews, each center employs an average of 10–15 doctors, with total medical staff ranging from approximately 460 to 690 per institution. We obtained 196 valid questionnaires from rural institutions and 263 from urban ones. Among all respondents, 258 were female, accounting for 56.21% of the total sample. The average age of participants was 40.87 years. In addition, 81.70% of doctors held a bachelor’s degree or above, and their average length of clinical practice was 16.88 years.

### 4.2. Hypothesis Testing Results

#### 4.2.1. Analysis Result of the Direct Association Between Doctor Empathy and Doctor-Rated Patient Adherence

The results of the model fit indices were: χ^2^ = 21.96, df = 10, *p* = 0.015 < 0.05, χ^2^/df = 2.20 < 5, GFI = 0.99 > 0.90, AGFI = 0.96 > 0.90, RMSEA = 0.05 < 0.08 (LO90 = 0.02, HI90 = 0.08), NFI = 0.96 > 0.90, CFI = 0.98 > 0.90, PGFI = 0.28 < 0.50. With the exception of PGFI falling below the 0.50 cutoff, all remaining indices met or surpassed the recommended thresholds, indicating marginally acceptable model fit. The standardized path coefficient reflecting the correlational linkage between doctor empathy and patient adherence was 0.17 (*p* = 0.009), which was statistically significant (Table 1 and Figure S2 in the Supplementary File).

#### 4.2.2. Analysis Result of the Mediation Role of Emotional Energy

Bootstrap analysis with 5000 resamples yielded the following model fit indices: χ^2^ = 129.01, df = 28, *p* < 0.001, χ^2^/df = 4.61 < 5, GFI = 0.95 > 0.90, AGFI = 0.89 approaching 0.90, RMSEA = 0.09 > 0.08 (LO90 = 0.07, HI90 = 0.11), NFI = 0.92 > 0.90, CFI = 0.93 > 0.90 and PGFI = 0.41 < 0.50. Except for AGFI and RMSEA close to the cutoffs, all other indices met or exceeded thresholds, suggesting marginally acceptable fit. The direct path from doctor empathy to adherence was significant (standardized path coefficient = 0.15, *p* < 0.05), but the path from emotional energy to adherence was not (standardized path coefficient = 0.06, *p* > 0.05). The indirect effect via emotional energy was non-significant (standardized path coefficient = 0.02, *p* > 0.05), thus failing to support hypothesis H2. (see Table 2, and Figure S3, Table S10 in the Supplementary File).

#### 4.2.3. Analysis Result of the Mediation Role of Doctor Treatment Behavior

The model was refined by establishing a covariance between error terms e16 and e17, according to modification indices (MI). Following this adjustment, bootstrapping with 5000 iterations yielded the following model fit indices: χ^2^ = 550.62, df = 135, *p* < 0.001, χ^2^/df = 4.08 < 5, GFI = 0.88, close to 0.90, AGFI = 0.84, close to 0.90, RMSEA = 0.082 > 0.08 (LO90 = 0.075, HI90 = 0.089), NFI = 0.92 > 0.90, CFI = 0.94 > 0.90, PGFI = 0.63 > 0.50. With the exception of GFI, AGFI, and RMSEA close to the recommended thresholds, all remaining fit indices met or exceeded the cutoffs, which indicates marginally acceptable model fit. The direct path from doctor empathy to adherence was significant (standardized path coefficient = 0.42, *p* < 0.01), but the path from treatment behavior to adherence was not (standardized path coefficient = 0.05, *p* = 0.367). The indirect effect was non-significant (standardized path coefficient = 0.02, *p* > 0.05), rejecting hypothesis H3. (See Table 3, Figure S4, and Table S11 in the Supplementary File).

#### 4.2.4. Analysis Result of the Chain Mediating Role of Emotional Energy and Doctor Treatment Behavior

The model was adjusted based on modification indices (MI), which allowed for correlations between e16 and e17, as well as between e9 and e10. Results from 5000 bootstrap iterations indicated the following model fit indices: χ^2^ = 754.25, df = 184, *p* < 0.001, χ^2^/df = 4.10 < 5, GFI = 0.86, close to 0.90; AGFI = 0.80, close to 0.90; RMSEA = 0.082, close to 0.08 (LO90 = 0.076, HI90 = 0.088); NFI = 0.91 > 0.90; CFI = 0.93 > 0.90; and PGFI = 0.62 > 0.50. With the exception of GFI, AGFI, and RMSEA close to the recommended thresholds, all remaining fit indices met or exceeded the cutoffs, which indicates marginally acceptable model fit. The direct path from doctor empathy to adherence was significant (standardized path coefficient = 0.16, *p* = 0.019), but paths from emotional energy (standardized path coefficient = 0.04, *p* = 0.657) and treatment behavior (standardized path coefficient = 0.02, *p* = 0.870) to adherence were not. The indirect chain effect was non-significant, so hypothesis H4 was not supported. (see Table 4, and Figure S5, Table S12 in the Supplementary File).

## 5. Discussion

### 5.1. Interaction Ritual Chains Theory Provides a Preliminary Conceptual Lens for Exploring the Empathy–Adherence Relationship, Though Further Investigation Is Needed

This study developed research hypotheses and a model grounded in Interaction Ritual Chains (IR Chains) to examine the correlational linkages between doctor empathy and doctor-rated patient adherence. Structural equation modeling was used to verify the proposed direct, mediating, and chain mediating pathways. The results of the analysis indicated that hypothesis H1: doctor empathy is positively correlated with doctor-rated patient adherence was supported. The standardized regression coefficient was 0.17 (*p* = 0.009). However, hypothesis H2: emotional energy is hypothesized to mediate the correlational linkage between doctor empathy and doctor-rated patient adherence, hypothesis H3: doctor treatment behavior is hypothesized to mediate the correlational linkage between doctor empathy and doctor-rated patient adherence, and hypothesis H4: emotional energy and treatment behavior are hypothesized to mediate the correlational linkage between doctor empathy and doctor-rated patient adherence were not supported. These findings suggest that IR chains are applicable and have explanatory value in understanding the association of doctor empathy with patient adherence in primary care clinical contexts.

The non-significant mediating roles of emotional energy and treatment behavior suggest that the indirect pathways specified by IR Chains were not empirically confirmed in this clinical sample. These mixed results carry nuanced theoretical implications, which should be interpreted with caution. On the one hand, the validated direct association corroborates the core logic of IR chains theory: doctors’ empathic responses during medical consultations constitute positive interaction rituals that are positively associated with patients’ willingness to follow treatment regimens. And doctor empathy shows a positive correlational linkage with doctor-rated patient adherence. Relevant studies have demonstrated that doctors’ expressions of empathy, including identifying patients’ psychological states through active listening and observation of verbal and non-verbal cues; delivering disease, diagnosis, treatment and management information to patients with patience and clarity; recognizing and alleviating patients’ negative emotions through reassurance and guidance; and addressing patients’ concerns, needs or fears about their conditions, positively related to patients’ treatment adherence [51,52,53,54]. This finding validates the basic explanatory capacity of IR chains theory for the empathy–adherence linkage in routine primary care practice.

On the other hand, the non-significant mediating and chain mediating role signal critical boundary conditions limiting the full explanatory power of IR chains theory in this study’s setting, which restricts the theoretical generalizability of the originally hypothesized multi-path mechanism. Two contextual factors may account for the insignificant indirect pathways. Firstly, doctor empathy has a direct association with patient adherence, which may overshadow indirect pathways. Second, consistent with Collins’ original IR Chains theory, emotional energy decays over time [11]. During interviews, many doctors reported high work enthusiasm early in their careers, but this energy gradually stabilized at moderate levels without obvious fluctuation. Additionally, heavy outpatient workloads restrict doctors to brief, superficial reminders during routine consultations, such as “maintain a light diet”, “reduce salt intake”, “eat more vegetables”, and “increase physical activity”. Compared with deliberate, targeted empathetic communication and sustained patient-centered clinical behaviors (e.g., regular community blood pressure and blood glucose screenings, medication dispensing, and follow-up home visits delivered as part of family doctor services), the routine brief advisory behaviors measured in this study exerted relatively weak predictive effects on patient adherence.

In conclusion, our study’s theoretical contributions must be framed cautiously in light of the incomplete support for the hypothesized IR chains-based mechanism. While the study confirms a direct empathy–adherence relationship consistent with IR chains theory, it cannot supply empirical evidence to validate the sequential indirect mediation pathways built from the theory. Accordingly, subsequent theoretical deductions and practical recommendations derived from this model will be refined to strictly align with the actual empirical results, avoiding overstated claims regarding the chain mediating mechanism of emotional energy and doctor treatment behavior.

### 5.2. Doctor’s Guidance Can Help to Facilitate the Disease Treatment

The findings of this study reveal that doctor empathy can directly encourage patient adherence. This suggests that improving doctors’ communication skills and empathy may be associated with more effective clinical diagnosis and treatment management by demonstrating greater understanding and compassion towards patients. In medical education and doctors’ training, in addition to focusing on improving doctors’ professional knowledge and skills, it is also necessary to focus on improving soft skills such as humanistic care and professionalism. Compared with professional knowledge and skills, medical humanities and professionalism usually receive little attention in medical education or medical-related vocational training [55]. Interviews with participating doctors revealed that many hold incomplete understandings of clinical empathy; some also argue that medical humanities interventions appear abstract to patients if they do not directly alleviate physical discomfort or address core health demands. As public living standards rise, patients no longer merely seek disease relief or cure—they also demand positive medical experiences and high-quality, humanized clinical services [26]. Medical practitioners who integrate rigorous clinical skill with strong moral standards consistently gain higher recognition and trust from patients.

The public also holds higher and more critical expectations of doctors’ medical ethics compared to other professional categories. Most patients with hypertension and diabetes are elderly adults with limited comprehension of professional medical knowledge, including treatment precautions and medical insurance policies, which require concise, patient explanations from doctors. This challenge is amplified among rural elderly patients with low educational background, who rely on repeated, simplified explanations of treatment regimens and policy rules. Without clear, effective communication, patients may misunderstand clinical advice, generate dissatisfaction, and experience deterioration in the doctor–patient relationship. Many young doctors interviewed reported communication barriers when interacting with elderly patients, stemming from limited clinical experience. An effective solution to bridge this gap is mentorship pairing: senior primary care doctors with extensive experience in chronic disease consultation can provide systematic guidance to junior practitioners. Senior mentors can share practical communication strategies for elderly populations and demonstrate how to translate complex medical terminology into plain, accessible language for patients. This hands-on mentorship approach delivers stronger real-world utility than purely theoretical classroom training [56]. Meanwhile, primary healthcare institutions can develop specialized training programs focused on clinical empathy and chronic disease communication, covering emotional recognition, simplified disease education, and psychological counseling tailored to hypertension and diabetes patients. Nevertheless, these practical suggestions remain preliminary. Controlled intervention trials are required to verify whether such training effectively elevates patient adherence rates by doctors, as the cross-sectional design of this study cannot establish causal intervention effects.

### 5.3. Strength and Limitation

This study innovatively applies IR Chains theory to the medical domain to explore the pathways linking doctor empathy to patient adherence in primary care and chronic disease consultations. The empirical results offer actionable references for improving doctor empathy competency and optimizing chronic disease management. Consistent with theoretical expectations, the cross-sectional analysis confirms a statistically significant direct association between doctor empathy and doctor-rated patient adherence, laying a foundational empirical basis for follow-up theoretical and practical research on doctor–patient interaction.

Despite the above contributions, this work has several limitations, which point to clear directions for subsequent research. First, the study adopts a cross-sectional design, which cannot draw rigorous causal conclusions. The hypothesized indirect pathways through emotional energy and doctor treatment behavior failed to receive statistical support and require repeated testing in future research. To resolve this issue, future scholars can refine the theoretical framework by prioritizing core influencing factors and expand the research scope to cover multi-level medical institutions and diverse participants. For instance, follow-up research can collect patient self-reported data (patient perception of doctor empathy and clinical behaviors) to match doctor-rated data; researchers can also record real doctor–patient consultations and invite trained third-party observers to score clinical behaviors via standardized observation scales. Such multi-angle data collection can effectively mitigate common method bias stemming from the single-source doctor self-reporting used in this paper. In addition, more rigorous longitudinal and experimental designs are recommended, including cohort studies, intervention-controlled trials, and matched doctor–patient paired surveys. These designs allow in-depth verification of the hypotheses, inter-variable correlations, causal mechanisms, feedback effects, and other outcome dimensions proposed by IR Chains theory, offsetting the inherent defect of cross-sectional data in causal inference. Second, this study only examines four core variables (doctor empathy, emotional energy, doctor treatment behavior, doctor-rated patient adherence) while ignoring multi-level contextual confounders. A wide range of micro, meso and macro factors may interfere with the empathy–adherence relationship, such as artificial intelligence medical tools that reshape doctor–patient communication, family dynamic characteristics, social support networks, and healthcare policy constraints. Although basic demographic attributes were set as control variables in this analysis, future models need more complex statistical techniques to capture the intricate interactive relationships among multiple real-world influencing factors. Third, most measurement tools in this paper are self-developed, which restricts the generalizability of the research findings. Only the doctor empathy scale was a mature translated scale; the questionnaires for emotional energy, doctor treatment behavior, and doctor-rated patient adherence were self-designed, given the scarcity of existing IR Chains-based measurement tools in medical research. Correspondingly, several structural models showed borderline fit, with RMSEA values exceeding 0.08 and GFI/AGFI indices falling below 0.90. These self-developed instruments are merely exploratory measuring tools and demand further cross-regional, cross-sample reliability and validity verification. Subsequent research can expand sampling to primary, secondary, and tertiary hospitals across diverse regions to revise and optimize the scales and replicate the research results. Fourth, this study relies on a single self-rated item for doctors to judge patients’ overall adherence, which is prone to subjective deviation and insufficient measurement validity. Future matched surveys could adopt validated multi-item patient self-adherence scales and incorporate objective indicators (e.g., medication refill records, follow-up rates, medication possession ratio, pill counts, smart bottle monitoring) to construct multidimensional adherence measures, reducing single-source reporting bias. Fifth, the questionnaire measuring doctor treatment behavior has conceptual limitations. The current questionnaire heavily focuses on doctors’ disease education work, yet patients’ disease knowledge does not always translate into actual treatment adherence. Subsequent measurement revisions can incorporate validated indicators of autonomy-supportive clinical behaviors from the existing literature to remedy this narrow conceptual coverage and optimize the measurement framework of doctor treatment behavior.

## 6. Conclusions

The results of the analysis revealed that only the hypothesis proposing a direct positive concurrent correlational linkage between doctor empathy and doctor-rated patient adherence was supported. Hypotheses concerning the mediating role of emotional energy and doctor treatment behavior, and their chain mediation in this relationship, were not statistically validated. These findings suggest that empathy and communication skills may be relevant to doctor-rated adherence, although intervention studies are needed to determine whether improving these skills leads to better adherence. Meanwhile, subsequent studies should expand the research scope, adopt diverse samples, and refine measuring instruments to verify the research hypotheses and theoretical model.

## Figures and Tables

**Table 1:** The regression coefficients of the direct association between doctor empathy and doctor-rated patient adherence.

Pathways	Estimated Values ^a^	Estimated Values ^b^	S.E.	C.R.	*p*
Doctor empathy—Doctor-rated Patient adherence	0.09	0.17	0.03	2.63	0.009
Age—Doctor-rated Patient adherence	−0.16	−0.08	0.09	−1.68	0.094
Gender—Doctor-rated Patient adherence	0.002	0.02	0.01	0.31	0.759
Marital status—Doctor-rated Patient adherence	0.16	0.06	0.16	1.01	0.311
Educational level— Doctor-rated Patient adherence	0.17	0.07	0.13	1.31	0.190

Note: C.R. (critical ratio) corresponds to the t-value, and S.E. indicates the standard error; ^a^: estimates reflect unstandardized path coefficients, ^b^: estimates reflect standardized path coefficients.

**Table 2:** The analysis results of the mediating role of emotional energy.

Pathways	Standardized Path Coefficients	Bias-Corrected 95% CI			Percentile 95% CI		
		Low	High	*p*	Low	High	*p*
Total effect	0.17	0.03	0.30	0.013	0.04	0.30	0.011
Direct effect: Doctor empathy— Doctor-rated Patient adherence	0.15	0.001	0.295	0.049	0.001	0.295	0.048
Indirect effect: Doctor empathy— Emotional energy— Doctor-rated Patient adherence	0.02	−0.03	0.07	0.338	−0.02	0.07	0.323

Note: Bias-Corrected and Percentile are two methods for estimating confidence intervals.

**Table 3:** The analysis results of the mediating role of doctor treatment behavior.

Pathways	Standardized Path Coefficients	Bias-Corrected 95% CI			Percentile 95% CI		
		Low	High	*p*	Low	High	*p*
Total effect	0.18	0.05	0.31	0.004	0.05	0.31	0.004
Direct effect: Doctor empathy—Doctor-rated Patient adherence	0.16	0.01	0.31	0.036	0.01	0.31	0.035
Indirect effect: Doctor empathy—Doctor treatment behavior—Doctor-rated Patient adherence	0.02	−0.03	0.07	0.426	−0.03	0.07	0.383

Note: Bias-Corrected and Percentile are two methods for estimating confidence intervals.

**Table 4:** The analysis results of the chain mediation role of emotional energy and doctor treatment behavior.

Pathways	Unstandardized Path Coefficients	Bias-Corrected 95% CI			Percentile 95% CI		
		Low	High	*p*	Low	High	*p*
Total effect	0.08 (standardized value: 0.18)	0.02	0.18	0.004	0.02	0.18	0.005
Direct effect: Doctor empathy— Doctor-rated Patient adherence	0.07 (standardized value: 0.16)	0.004	0.181	0.036	0.004	0.180	0.036
Total Indirect effect	0.01 (standardized value: 0.02)	−0.02	0.03	0.43	−0.02	0.03	0.43
(a) Doctor empathy— Emotional energy—Doctor-rated Patient adherence	0.007	−0.03	0.04	0.699	−0.03	0.04	0.637
(b) Doctor empathy— Doctor treatment behavior—Doctor-rated Patient adherence	0.001	−0.02	0.01	0.850	−0.02	0.01	0.872
(c) Doctor empathy— Emotional energy-Doctor treatment behavior— Doctor-rated Patient adherence	0.002	−0.03	0.03	0.860	−0.03	0.03	0.872

Note: Bias-Corrected and Percentile are two methods for estimating confidence intervals.

## Data Availability

The datasets generated during and/or analyzed during the current study are available from the corresponding author on reasonable request.

## Supplementary Materials

The following supporting information can be downloaded at: https://www.mdpi.com/article/10.3390/healthcare14152378/s1, Figure S1: Interaction Ritual Chains; Figure S2: Direct association between doctor empathy and doctor-rated patient adherence; Figure S3: Mediation role of emotional energy; Figure S4: Mediating role of doctor treatment behavior; Figure S5: Mediation role of emotional energy and doctor treatment behavior; Table S1: Demographic character of participants; Table S2: Initial concepts derived from open coding of interview data; Table S3: Corresponding categories; Table S4: Main categories; Table S5. Core categories; Table S6: References of designing questionnaires; Table S7: CRs, and AVEs of Chinese version of JSPE-HP; Table S8: CRs, and AVEs of doctor emotional energy questionnaire; Table S9: CRs, and AVEs of doctor treatment behavior questionnaire; Table S10: The regression coefficients of the mediating role of emotional energy; Table S11: The regression coefficients of the mediating role of doctor treatment behavior; Table S12: The regression coefficients of the chain mediating role of emotional energy and doctor treatment behavior; Table S13: Results of exploratory factor analysis for the doctor measurements.
