# Supplementary material for: Correlational Linkages Between Doctor Empathy and Doctor-Rated Patient Adherence: An Interaction Ritual Chains Perspective
Source: Healthcare (Basel). 2026 Aug 4;14(15):2378. doi: 10.3390/healthcare14152378 (PMC13466128; doi:10.3390/healthcare14152378)
Supplement: Supplementary file 1 [file healthcare-14-02378-s001.zip › healthcare-4384313-supplementary.pdf]

Supplementary

S1.Interaction Ritual Chains

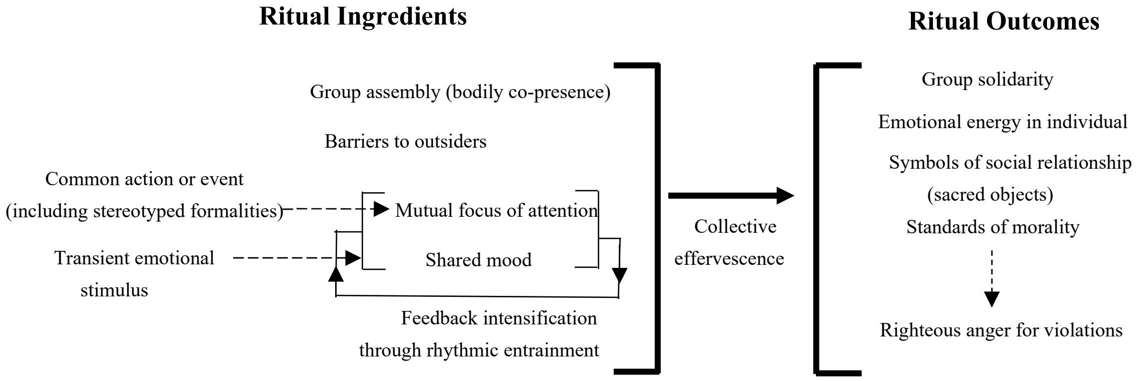

Figure S1. Interaction Ritual Chains
(Reference: Randall C. Interaction Ritual Chains; Lin J, Wang P, Song L, Trans.; Commercial Press: Beijing, China, 2009. (In Chinese))

Other elements mentioned in the ritual chains, such as mutual focus and shared emotional states, are reinforced through rhythmic, solidarity-based feedback. This is the process by which interaction participants become increasingly engrossed in their joint actions, more aware of each other's actions and feelings, and more attuned to each other's consciousness. As the interaction becomes more engaging, participants become immersed in the rhythm and atmosphere of the conversation, experiencing their shared emotions more intensely. Collective excitement is a shared emotional experience among the participants of an interaction, representing the accumulation of shared emotions to a certain degree. For example, cheering crowds become more fervent, participants in religious ceremonies become more reverent and solemn, and mourners at funerals become more sorrowful, often with a transient or momentary nature. According to everyday expressions, rituals are often described as formal ceremonies conducted through a set of procedural actions, such as recitations, singing, wearing traditional attire, making introductory remarks or providing emotional guidance. Rituals become successful when participants genuinely experience shared emotions and clearly understand each other's consciousness; otherwise, they remain mere formalities or rigid ritualism. Additionally, some non-procedural activities or spontaneously formed rituals can also provide participants with a common focus and shared emotional experiences. Therefore, the key elements of rituals are not the common behaviors or events, nor the transient emotional stimuli, but rather the auxiliary role they play in fostering a mutual focus and shared emotions.

S2.Authorization of JSPE-HP

2022/9/12 打印邮件

主　题:	RE: Consultation about JSPE-HP again_shumin mai	
发件人:	"Jefferson Scale of Empathy" <empathy@jefferson.edu>	2022-5-17 20:18:37
收件人:	"Shumin Mai-Zhejiang University" <shumin_mai@zju.edu.cn>	
抄　送:	"Mohammadreza Hojat" <Mohammadreza.Hojat@jefferson.edu>, "Aaron Douglas" <Aaron.Douglas@jefferson.edu>	
附　件:	Jefferson Chinese version linguistic changes_shumin.pdf	

Dear Shumin,

Thank you very much for your patience in working with us on confirming the translation for your project. After careful review from our colleague, it was determined that the translation you modified is the better of the two, yours being more smooth and closest to the English. Please feel free to use this in your project. We will be using this one when future researchers ask to use a Chinese traditional HP-version. Please be sure to also include the copyright line.

If any other researcher wants to use the translated scale; again, permission to use any translation of the JSE may be given only by us. Please have them contact us. Do not share the files with anyone who is not part of this project.

Good luck with your project!

Kind regards,

Shira Carroll

Empathy Project Coordinator | Assessment Scales

Asano-Gonnella Center for Research in Medical Education & Health Care

From: Shumin Mai-Zhejiang University <shumin_mai@zju.edu.cn>
Sent: Tuesday, May 10, 2022 8:55 PM
To: Jefferson Scale of Empathy <empathy@jefferson.edu>
Cc: Mohammadreza Hojat <Mohammadreza.Hojat@jefferson.edu>
Subject: Re: Consultation about JSPE-HP again_shumin mai

S3. Development of doctor emotional energy and doctor treatment behavior questionnaires

S3.1 Method

(1) Participants

The Questionnaires will be developed based on the qualitative interview with Doctors. General practitioners, Traditional Chinese Medicine doctors, and village doctors who employed in medical institutions with independent prescription rights, routinely treat patients with hypertension and/or diabetes will be invited to participate in the interview. Based on research feasibility and data availability, qualitative interviews are planned in one or two districts in Zhejiang Province, where cities and towns are concentrated. Interviews with primary-level healthcare institutions will be conducted in Yuhang (one community healthcare center) and Shangcheng (one community healthcare center) in Hangzhou, Kaihua (two township healthcare centres) in Quzhou, and Suichang (three township healthcare centres) in Lishui. Meanwhile, we also conduct the interview in a tertiary hospital with a strong reputation for comprehensive medical care, extensive service coverage and a long history of general medicine development in Zhejiang Province. We conducted interviews there in order to compare the potential differences between primary-level healthcare centers and the tertiary hospitals. We plan to invite 10-20 doctors from each of the selected medical institutions, and the planned interview time for each interviewee is 20-30 minutes.

(2) Method

The interview outline will be designed, and during the interview, doctors will be consulted about their views on the influence of doctor empathy on patient adherence and the doctors themselves. According to the grounded theory,[1] the interview materials will be coded and analyzed using a three-stage process by Nvivo 12.0 software: consisting of open, axial and selective coding. In order to explore the major content and construction of the questionnaires. The items will be developed based

on the interview data analysis, relevant research and discussion among the research teams.

S3.2 Results

(1) Demographic character of participants

The interviews were conducted between June and August 2022. A total of 45 doctors took part in the study. Of these, 28 (62.22%) were from urban areas, 17 (37.78%) were from townships, 40 (88.89%) were from primary-level healthcare institutions, 37 (82.22%) were general practitioners, 23 (51.11%) were male and 22 (48.89%) were female. The majority (62.22%) had a bachelor's degree or above, and most (82.22%) had worked for ten years or more. (Table S1)

Table S1. Demographic character of participants

Variables		Frequency	Percentage（%）
Areas	Cities	28	62.22
	Town/villages	17	37.78
Healthcare institutions	Primary-level	40	88.89
	Tertiary hospitals	5	11.11
Doctor types	General practitioners	37	82.22
	Traditional Chinese medicine practitioners	5	11.11
	Village doctors	3	6.67
Gender	Male	23	51.11
	Female	22	48.89
Age	18-49	31	68.89
	≥ 50	14	31.11
Educational level	Below bachelor	17	37.78
	Bachelor and above	28	62.22
Working year	<10	8	17.78
	≥ 10	37	82.22

(2) Interview data analysis result

Based on responses and opinions from doctors to the question, 'What roles or influence do you believe doctor empathy? For example, what positive or negative influence does it have on the doctor themselves?' A three-level coding process was performed, resulting in 16 initial concepts, 15 corresponding categories, 14 main categories and four core categories (Tables S2, S3, S4, and S5).

Table S2. Initial concepts derived from open coding of interview data

	Initial concepts	Representative original sentences
1	Patients treat their illnesses as directed/suggested by their doctors	We shouldn't neglect good communication just because the outpatient volume is high. Good communication can improve patient treatment.(Doctor's quote)
2	Blood pressure and blood sugar are controlled/blood pressure and blood sugar lowering effects are achieved, and the condition improves	Doctors' empathy improves the adherence of patients with "two chronic diseases", thereby improving the treatment effect, increasing patients' trust in doctors, satisfaction, and better medical experience. (Doctor's Quote)
3	Patients trust doctors, share personal information with doctors/regard doctors as family members	1. Doctors who patiently explain things to their patients can gain their trust and recognition. (Doctor's quote)<br>2.Doctors' empathy improves the adherence of patients with "two chronic diseases", thereby improving the treatment effect, increasing patients' trust in doctors, satisfaction, and better medical experience. (Doctor's Quote)<br>3. If you communicate well with your patients, for example, they will think of you as a family member, trust you and open up to you. This is what it means to be a true family doctor — a member of the family. When they have a problem, you are the first person they think of. (Doctor's quote)
4	Patients are satisfied with the services provided by doctors	Communicating with patients more often and making more eye contact will make them feel much better and more comfortable. (Doctor's quote)
5	Patient acknowledgement/thank you to the doctor	Some ordinary people realise that you are responsible and conscientious at work, and they are very grateful to you. You may even hear patients say, 'Oh, your family doctor is so kind to us.' (Doctor's quotes)
6	Friendly relationship between doctors and patients	Doctors need to communicate more with their patients. When doctors make an effort for their patients, the patients will feel that they are truly doing good for them, and the relationship between doctors and patients will naturally improve. (Doctor's quote)
7	Feeling irritable, tired, or sad	1. Doctors who are exposed to too much negativity from their patients may also become negative themselves. Sometimes, being too sympathetic to a patient can be sad, too. (Doctor's quote)<br>2. Patients with mental health conditions such as anxiety and depression often want to communicate with their doctors. They often want to chat with me. I have to see the next patient, but they are still waiting for me to find time to talk to them again. Sometimes I find this very annoying, but I can't scold them or show impatience. I also need to control my emotions. Sometimes, after working for a long time in the morning, I am exhausted and in a bad mood. Sometimes patients come in, ask about everything and disrupt my consultation with other patients, then I will get very annoyed at this situation. (Doctor's quote)

Table S2. Initial concepts derived from open coding of interview data (continuous)

	Initial concepts	Representative original sentences
8	Degree of being affected by the patient's emotions/maintaining objectivity	1. When we visit patients, they often talk to us about many different things, but we are generally not affected by their emotions. (Doctor's quote)<br>2. I won't be affected by patients' negative or pessimistic emotions. Instead, I should offer them comfort and not allow myself to be distracted by their thoughts. I should guide them slowly. Elderly people sometimes need encouragement and good advice. In general, I believe that we should offer more comfort while remaining objective and calm and not allowing ourselves to be affected by their emotions. (Doctor's quote)
9	Doctors are active and proactive in their work.	It is a real sense of accomplishment to have so many patients trust me, so I get up to work early every morning to ensure that patients can get their medication and medical appointments earlier. (Doctor's quote)
10	Job satisfaction/feeling of accomplishment	It's a great sense of accomplishment to have so many patients trust me. (Doctor's Quote)
11	Job responsibilities/duties	1. As primary-level healthcare providers, we should be available to patients at any time. If a patient calls us for help with measuring their blood pressure or blood sugar, for example, we should visit them and should not refuse. (Doctor's quote)<br>2. The interviewed doctors were able to understand and empathize with their patients, meeting their needs and doing their jobs well. During the epidemic, they took risks to treat patients with limited mobility and provided home care after work based on patient needs, demonstrating their sense of responsibility. (Interview memo)
12	Doctors are passionate about their work/work with enthusiasm; doctors become numb after working for a long time	Young doctors are often enthusiastic when they first start working, but this tends to wane over time. This is mainly because they don't fully understand their role. I think you should love your job, then the more you do, the more enthusiastic you become. You need to adjust your mindset and think positively. Some people who become less enthusiastic over time will eventually see fewer and fewer patients because their mindset can affect their actions. (Doctor's quote)
13	Feel sympathy or pity for the patient	Frequent exposure to patients' negative emotions will have a certain impact on yourself, and feel pity for the patient. (Doctor's Quote)
14	Patiently and actively guide patients/comfort patients	When I encounter patients who are expressing negative emotions, I comfort and counsel them patiently, guiding them to think more positively. (Doctor's quote)
15	Solve patients' medical needs/demands	At primary-level clinics, we should make ourselves available to patients. If a patient calls and asks us to take their blood pressure or blood sugar levels, we should be happy to help and should not refuse. (Doctor's quote)
16	Repeated reminders	Many elderly people have hypertension and diabetes, and some have poor comprehension skills. It is important to explain the consequences of their illness. Be patient, explain things several times and repeat them next time. Repeated reminders can help improve treatment adherence. (Doctor's quote)

Table S3. Corresponding categories

	Corresponding categories	Initial concepts
1	Patients follow the doctor's advice.	Patients treat their illnesses as directed/suggested by their doctors
2	Effective control of two chronic diseases	Blood pressure and blood sugar levels are controlled, and lowering effects are achieved, improving the physical condition.
3	The level of trust that patients have in their doctors	Patients trust doctors, share personal information with them and regard them as family members.
4	Improve patient satisfaction	Patients are satisfied with the services provided by doctors
5	Patients appreciate/recognize doctors	Patient thanks/thanks to the doctor
6	Harmonious relationship between doctors and patients	Friendly relationship between doctors and patients
7	Emotional contagion	Degree of being affected by the patient's emotions/maintaining objectivity; irritability, fatigue, sadness.
8	Work enthusiasm	Doctors are active and proactive in their work
9	Job satisfaction/fulfillment	Job satisfaction/feeling of accomplishment
10	Doctors' sense of responsibility	Job responsibilities/job duties
11	Doctors' passion/commitment to their work	Doctors are passionate about their work/work with enthusiasm; doctors become numb after working for a long time
12	Sympathy for patients	Feel sympathy or pity for the patient.
13	Doctor comforts patient	Patiently and actively guide patients/comfort patients
14	Doctors assist in solving patients' problems	Solve patients' medical needs/demands
15	Doctor explains patiently	Repeated reminders

Table S4. Main categories

	Main categories	Corresponding categories
1	Patient treatment adherence	Patients follow/adopt doctor's advice
2	Disease treatment effects	Effective control of two chronic diseases
3	Patient trust doctor	The level of trust that patients have in their doctors.
4	Patient satisfaction	Improve patient satisfaction
5	Patient gratitude	Patients appreciate/recognize doctors
6	Harmonious doctor-patient relationship	Harmonious relationship between doctors and patients
7	Doctor's emotions	Emotional contagion
8	Doctors' work initiative and positivity	Work enthusiasm
9	Doctor's sense of accomplishment	Job satisfaction/fulfillment
10	Doctor's sense of responsibility	Doctors' sense of responsibility
11	Work enthusiasm	Doctors' passion/commitment to their work
12	Doctors sympathize with patients	Sympathy for patients
13	Communication between doctors and patients	Doctor comforts patient, Doctor explains patiently
14	Solving patient problems	Doctors assist in solving patients' problems

Table S5. Core categories

	Core categories	Main categories
1	Patient disease treatment behavior	Patient treatment adherence
2	Quality of medical services	Disease treatment effects; Patient trust doctor； Patient satisfaction; Patient gratitude; Harmonious doctor-patient relationship
3	Doctor emotional energy	Doctor's emotions; Doctors' work initiative and positivity; Doctor's sense of accomplishment; Doctor's sense of responsibility; Work Enthusiasm; Doctors sympathize with patients
4	Doctor treatment behavior	Communication between doctors and patients; Solving patient problems

In general, according to the interview data analysis, doctor empathy mainly influence patient treatment adherence, medical quality, doctor emotional energy and doctor treatment behavior, which are corresponding to our research model base on IRs.

(3) Primary draft of the questionnaires

Base on the analysis result, relevant research and discussion among research group, we developed the primary draft of the questionnaires. Table demonstrated the reference of questionnaire design. And the content of the questionnaires is descripted in the section 4. (Table S6)

Table S6. References of designing questionnaires

Variables	References
Doctor emotional energy	Work enthusiasm-related questionnaires/scales [2-4], employee work engagement scales [5-6], and nursing staff responsibility questionnaire [7]
Doctor treatment behavior	Qualitative analysis result; and discussion among research group

Note: References of table S6:

1. Glesne, C. *Becoming qualitaitve researchers:An introduction(fifth edition);Cui M,Su J,Trans.*.Peking University Press, Beijing, China,2021.*(*in Chinese)

2.Carmeli, A., Ben-Hador, B., Waldman, D. A., & Rupp, D. E. (2009). How Leaders Cultivate Social Capital and Nurture Employee Vigor: Implications for Job Performance. *Journal of Applied Psychology*, *94*(6), 1553–1561. https://doi.org/10.1037/a0016429

3.Wu, S.A study on the work zest among primary and middle school teachers in the state sector of Hunan Province [Master’s thesis]. Nanchang University;2012. (in Chinese). Available from: https://oversea-cnki-net.ezproxy.lb.polyu.edu.hk/kcms2/article/abstract?v=f9AMVDUgxC-PU_xOTO2plNZwnQOwnxTDOEbPr-pLFnrEUiI64vB5gu76D5hDAB05m6d373Uf_AwOrEvgQIixWFDtZXUVYS8p2mhT04iW4XoKepOL_DZ3p1KvKqQohiIun62ckopCdbcnu4O84CZCtCWZo0t3KJgjWpAUr_ZXQk0=&uniplatform=OVERSEA

4.Zhai, L.A study on the relationship between public servant's identity and work enthusiasm:a moderated mediation model [Master’s thesis]. Lanzhou Univerisy;2021. (in Chinese) Available from:https://oversea-cnki-net.ezproxy.lb.polyu.edu.hk/kcms2/article/abstract?v=f9AMVDUgxC_mbZCF2BtGxCwDeAMl1qvD1tI-aNAWGhkw1tGWV01j0uAVugF6UWu4lD9CKZiK7sSbta_CvKCzbtFl00Ln5Z9-

rM9DdNG2Lw8PfvKYbt5zwqqnYAFQ600uWpwOqDSJZQaBYWPuEkvBqF47XPV5fy7s63 wZL6jWihA=&uniplatform=OVERSEA
5.Schaufeli, W. B., Salanova, M., González-romá, V., & Bakker, A. B. (2002). The Measurement of Engagement and Burnout: A Two Sample Confirmatory Factor Analytic Approach. *Journal of Happiness Studies*, *3*(1), 71–92. https://doi.org/10.1023/a:1015630930326
6.Zhang, Y.; Gan, Y. The Chinese version of Utrecht work engagement scale: An examination of reliability and validity. Chin. J. Clin. Psychol. 2005, 268–270+281. Available from: https://oversea-cnki-net.ezproxy.lb.polyu.edu.hk/kcms2/article/abstract?v=f9AMVDUgxC_cXg4j9eIe1A4sUYXjPq_xtzUx-IE-vpX_j9OOFQ5jsAoLjNjUUJXF2_YXbtIdd3KhWVvZYBrLC8sjasPSK2N7VlKDtpzvhbgOUZmMF6rft05Q2svAbFW939k4Pt0Z4Zg3wvQaPkRZkruUSnPBBN2Pg-&uniplatform=OVERSEA. (In Chinese)
7. Wu Y,Xu C,Chen X. Impact of primary nursing care on nurses' work responsibility. *Journal of nursing science*.2013; 28(07):40-41. Available from: https://oversea-cnki-net.ezproxy.lb.polyu.edu.hk/kcms2/article/abstract?v=f9AMVDUgxC-6X39EImWRw7e3V-BpjN8OzGt6hR4TkA3HtyHeGYLrrXCI26Am3b_KC4sTyCjyl9otkScMx2C5Ty7EAgFBLpotnlob-bb3d-rP-zTPrh4LTHBPDIf4umBG0m2CUxrHDZa7mU5IGt-atjZ8q9yDrXk9&uniplatform=OVERSEA.(in Chinese)

S4.The reliability and validity of the measurements in this study

S4.1 Method

(1) Participants

Doctors from the primary-level clinics in Zhejiang Province of China would be invited to participate in the online survey. The inclusion criteria were (1) employees of the clinics; (2) with independent prescription rights; (3) good at treating hypertension and (or) diabetes;(4) agree to participate and sign the informed consent form.

(2) Data management

We distributed 504 questionnaires, and 459 validated questionnaires were included into the data analysis.

(3) Statistical analysis

The reliability and validity of the Chinese version of JSPE-HP, doctor emotional energy questionnaire, and doctor treatment behavior questionnaire are evaluated. To test the internal consistency reliability, we calculated Cronbach's α values for the treatments. A Cronbach's α coefficient greater than 0.70 was considered satisfactory.[1,2] Composite reliability (CR) and convergent and discriminant validity were tested through confirmatory factor analysis (CFA). [1, 3] The CFA model was evaluated by goodness-of-fit index (GFI), adjusted GFI (AGFI), Normed fit index(NFI), and Comparative fit index(CFI), Parsimony goodness-of-fit index(PGFI), and root mean square error of approximation (RMSEA). When GFI, AGFI, NFI and CFI greater than 0.90, PGFI greater than 0.50 and REMEA less than 0.08 indicating the research model is acceptable. [3, 4] CR is used to evaluate the consistency of latent construct indicators, namely showing the degree about measurement indicators sharing the latent variables. A CR greater than 0.70 was considered satisfactory.[1, 3] The average variance extracted (AVE) means the variation degree to which latent

variables can explain the measurement indicators. It was used to determine convergent validity. When AVE reaches 0.50 or higher, the indicator variable can effectively reflect its latent variable.[3, 5, 6]

S4.2 Results

S4.2.1 The Chinese version of JSPE-HP

The Cronbach's alpha is 0.85, indicating that the Chinese version of Jefferson Scale of Physician Empathy for physicians and health professional (JSPE-HP) is internally consistent.

We set the CFA model based on the theoretical structure of the scale. The analysis result demonstrates that $\chi 2$ =491.37, df=224, χ^2/df=2.94, GFI=0.90 equal to 0.90, AGFI=0.87<0.90, and RMSEA=0.07 <0.08 with a 90% CI of 0.06 to 0.07, NFI=0.87<0.90, CFI=0.91, PGFI=0.71 >0.50. With the exception of AGFI and NFI closed to the recommended thresholds, all remaining fit indices met or exceeded the cutoffs, which indicates marginally acceptable model fit. Except item 1 and item 18, the regression weights of the other items were greater than 0.40. In terms of the CRs of perspective taking dimension, compassionate care dimension and stand in patient's shoes dimension were 0.88,0.81 and 0.74, respectively, all exceeding 0.70. The AVEs of those three dimensions (latent variables) were 0.43, 0.40 and 0.59, respectively. The dimension of stand in patient's shoes was above 0.50 while the other two dimensions were lower than 0.50. For assessing discriminant validity, a $\chi 2$ difference test was performed on the constrained model, which constrained the phi coefficient to equal 1, with a free model without this constraint. In all cases, the $\chi 2$ difference was significant, indicating satisfactory discriminant validity (Table S7).

(Note: In 2012, Chinese scholars Wen Deliang et al. [7] translated JSPE-HP into Chinese and validated its reliability and validity among clinical doctors. However, following the implementation of Jefferson University's updated authorization and registration system for the scale, their scale authorization needed to be assessed again. Jefferson University conducted a comparative linguistic review and concluded that the Chinese version of the JSPE-HP developed by our research team is better. Therefore, the university agrees and supports the use of it as a measure of doctor empathy in this study.

Table S7. CRs, and AVEs of Chinese version of JSPE-HP

Items	Standard loading	CR	AVE
Perspective taking		0.88	0.43
item2	0.59		
item4	0.66		
item5	0.52		
itme9	0.69		
item10	0.67		
item13	0.68		
item15	0.56		
item16	0.72		
item17	0.70		
item20	0.73		
Compassionate care		0.81	0.40
item1	0.25		
item7	0.70		

item8	0.70		
item11	0.80		
item12	0.75		
item14	0.77		
item18	0.05		
item19	0.58		
Stand in patient's shoes		0.74	0.59
item3	0.69		
item6	0.84		

Note: AVE: average variance extracted.

S4.2.2 Doctor emotional energy questionnaire

The primary draw of doctor emotional energy questionnaire was developed as three dimensions: sense of responsibility (6 questions), work enthusiasm (4 questions), and sympathy for patients' misfortune (2 questions). Utilizing a 5-point Likert scale anchored at 1 = "strongly disagree" to 5 = "strongly agree". Incomplete responses with missing values exceeding 20% of items (>3 unanswered items) will be excluded. Then the remaining missing data will be addressed through mean imputation.

The Cronbach's alpha of emotional energy questionnaire is 0.92. Then, the three-factor structure of the questionnaire was examined using confirmatory factor analysis (CFA). The analysis results of the model indices were :χ^2=375.59， df= 51， P<0.001， χ^2/df=7.36 >5， GFI= 0.88 near 0.90， AGFI= 0.81<0.90， RMSEA=0.12>0.08（LO90=0.11， HI90=0.13）， NFI=0.91>0.90， CFI=0.92>0.90， PGFI=0.57>0.50. With the exception of χ^2/df, GFI and AGFI closed to the recommended thresholds, all remaining fit indices met or exceeded the cutoffs, which indicates marginally acceptable model fit. Items analysis revealed that all measurement items demonstrated factor loadings >0.45, except for Item 7 (factor loading <0.45), which should be considered for removal. The composite reliability (CR) values for all three dimensions exceeded 0.50, indicating adequate internal consistency. Convergent validity was partially supported with average variance extracted (AVE) values: 0.43 for work enthusiasm (approaching 0.50 criterion), and values >0.50 for sense of responsibility and sympathy for patients' misfortune. (Tables S8).

The doctor emotional energy Questionnaire was found to be satisfactory in terms of its overall quality. In order to enhance the quality of the questionnaire, question 7 was eliminated. The final questionnaire comprised 11 items with three dimensions, including 6 items on sense of responsibility, 3 items on work enthusiasm, and 2 items on sympathy for patients' misfortune.

Table S8. CRs, and AVEs of doctor emotional energy questionnaire

**Items description**	**Standard loading**	**CR**	**AVE**
**Sense of responsibility**		0.92	0.67
1.daily treatment work will not be influenced by personal emotions	0.78		
2.try best to treat patients' illness	0.83		
3.maintain high level of work enthusiasm	0.86		
4.learn the advanced treatment skill and knowledge to cure patients' disease	0.88		
5.self-encourage to work hard and actively manage patients' illnesses	0.86		
10.still worry about patients' health after getting off	0.68		

**Work enthusiasm**		0.73	0.43
6.feeling energetic at work	0.83		
7.current job is boring and unfulfilling	0.25		
8.eager to go to work after waking up	0.66		
9.willing to communicate with patients and enjoy interacting with them	0.73		
**sympathy for patients' misfortune**		0.86	0.76
11.feel sorry for the patient's misfortune	0.85		
12.feel of tenderness and caring for patients who have difficult or unfortunate experiences in their lives	0.90		

Note: items 7 is reverse scoring

S4.2.3 Doctor treatment behavior questionnaire

The questionnaire, which was designed for the study in order to ascertain doctor treatment behavior, included a single dimension of 11 measurement questions. The questions are evaluated using a 5-point Likert scale (1 = strongly disagree, 5 = strongly agree). Data with missing responses exceeding 20% of the items (>3 items without answered by the participants) were excluded, and any remaining missing values were addressed using mean imputation.

The Cronbach's alpha of doctor treatment behavior questionnaire is 0.97. Confirmatory factor analysis was used to further test the fit of the single dimension structure of the doctor treatment behavior Questionnaire. The results of the analysis showed that the model fit indices were: χ^2=477.44, df=44, *P* <0.001,χ^2/df=10.85>5,GFI= 0.82<0.90,AGFI= 0.73<0.90, RMSEA=0.15>0.08(LO90=0.14，HI90=0.16)，NFI=0.93>0.90，CFI=0.93>0.90，PGFI=0.55>0.50. With the exception of χ^2/df, GFI, AGFI and RMSEA do not meet the recommended thresholds, all remaining fit indices met or exceeded the cutoffs, which indicates marginally acceptable model fit. Through the analysis, it was found that the factor loadings of the questions ranged from 0.77 to 0.94, all of which were greater than 0.45, and the reliability coefficients ranged from 0.60 to 0.87, all of which were greater than 0.20. The combined reliability of the whole questionnaire was 0.97, and the AVE value was 0.76, which indicated that the questionnaire had acceptable combined reliability and convergent validity. (Table S9) Thus, the final version of doctor treatment behavior questionnaire consisting of 11 items with a single dimension.

Table S9. CRs, and AVEs of doctor treatment behavior questionnaire

Items description	Standard loading	CR	AVE
total questionnaire		0.97	0.76
1.provide patients with clear explanations of the causes of hypertension (or) and diabetes, and the reasons for adopting appropriate treatment methods	0.77		
2.remind patients to take medication on time and in	0.90		

accordance with the dosage regularly	
3.remind patients to measure their blood pressure or (and) blood glucose regularly	0.92
4.remind patients to pay attention to a light diet and reasonable intake of vegetables and fruits regularly	0.94
5.remind patients to actively participate in physical exercise regularly	0.92
6.remind patients to quit smoking (or) and drinking regularly	0.90
7.remind patients to maintain a good mindset/emotion in their daily life regularly	0.90
8.remind patients the adverse effects of violating medical advice	0.90
9.provide comfort to patients when they have negative emotions such as anxiety, depression and worry.	0.84
10.try best to resolve the pain or difficulties caused by diseases to patients	0.78
11.reply patiently when patients make enquiries by phone or SMS	0.83

S4.3 Conclusion

In general, the Chinese version of JSPE-HP, doctor emotional energy and doctor treatment behavior questionnaires have acceptable quality.

References:

1. Yi, Y., & Gong, T. (2013). Customer value co-creation behavior: Scale development and validation. *Journal of Business Research*, *66*(9), 1279–1284. https://doi.org/10.1016/j.jbusres.2012.02.026
2. Ahmadizadeh, M. J., Ebadi, A., Sirati nir, M., Tavallaii, A., Sharif Nia, H., & Lotfi, M.-S. (2019). Development and psychometric evaluation of the Treatment Adherence Questionnaire for Patients with Combat Post-traumatic Stress Disorder. *Patient Preference and Adherence*, *13*, 419–430. https://doi.org/10.2147/PPA.S175353
3. Wu M. *Operation and application of Structural Equation Modeling (AMOS) (in Chinese)*. Chongqing University Press, Chonqing, China,2010.
4. Mazur, J., Zaborek, P. Validating DART model. *Int J Manag Econ*. 2014,*44*(1): 106-125.https://doi.org/10.1515/ijme-2015-0012
5. Hau, L. N., Tram Anh, P. N., & Thuy, P. N. (2017). The effects of interaction behaviors of service frontliners on customer participation in the value co-creation: a study of health care service. *Service Business*, *11*(2), 253–277. https://doi.org/10.1007/s11628-016-0307-4
6. Hsieh, Y.C., Chiu, H.C., Tang, Y.C., Lin, W.Y. Does raising value co-creation increase all customers' happiness? *J Bus Ethics*. 2018,*152*(4):1053-1067. doi:10.1007/s10551-016-3293-5\
7. Ma, X., Wen, D. Analysis on reliability and validity of Jefferson scale of physician empathy.*Practical Preventive Medicine*.2012,*19*(12):1779-1781. Available from : https://oversea-cnki-net.ezproxy.lb.polyu.edu.hk/kcms2/article/abstract?v=f9AMVDUgxC_0Yh1q2pLpMDQOlILDl5QKwQ7ujl

gMGz0urHHncVNbJVyk3ZDVvIubGf1IWV7RYglXvWXQOrzgf3f8HO-Dwf8x4PlWwWfqiOZfLHwF1Qi2wCCvQAfCGeAV5DwYraHjiVjVi8d_ocLiY1giDmaUNnKv&uniplatform=OVERSEA.(in Chinese)

S4.4 The analysis result of hypotheses

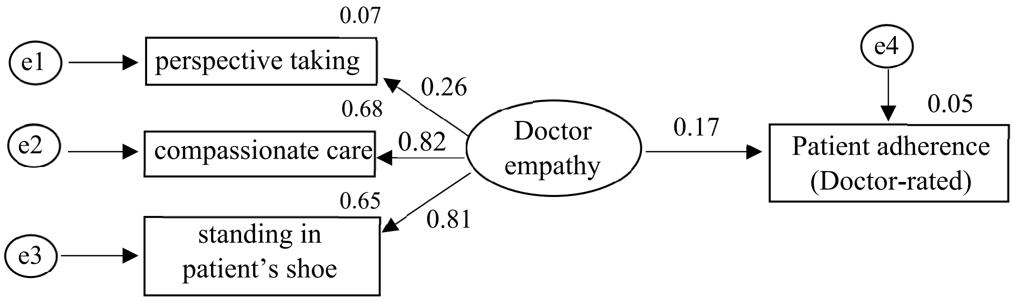

Figure S2. Direct association between doctor empathy and doctor-rated patient adherence

Note: The values shown in the figure are standardized estimates. Doctor's gender, age, marital status, and educational level were controlled during analysis. To maintain graphical clarity, the path analysis results are mainly showed the relationship between doctor empathy and doctor-rated patient adherence

Table S10. The regression coefficients of the mediating role of emotional energy

Pathways	Estimated values [a]	Estimated values [b]	S.E.	C.R.	*P*
Doctor empathy—Doctor-rated Patient adherence	0.07	0.15	0.03	2.28	0.023
Doctor empathy—Emotional energy	0.66	0.36	0.14	4.71	<0.001
Emotional energy—Doctor-rated Patient adherence	0.01	0.06	0.01	1.08	0.282
Gender—Emotional energy	0.25	0.03	0.36	0.69	0.489
Age—Emotional energy	0.07	0.17	0.03	2.98	0.003
Marital status—Emotional energy	-0.48	-0.04	0.61	-0.79	0.431
Educational level—Emotional energy	-0.21	-0.02	0.48	-0.43	0.667
Gender—Doctor-rated Patient adherence	-0.16	-0.08	0.09	-1.71	0.087
Age— Doctor-rated Patient adherence	0.0001	0.01	0.01	0.15	0.881
Marital status—Doctor-rated Patient adherence	0.17	0.06	0.16	1.05	0.293
Educational level—Doctor-rated Patient adherence	0.17	0.07	0.13	1.35	0.179

Note: C.R. (critical ratio) corresponds to the t-value, and S.E. indicates the standard error; a: estimates reflect unstandardized path coefficients, b: estimates reflect standardized path coefficients.

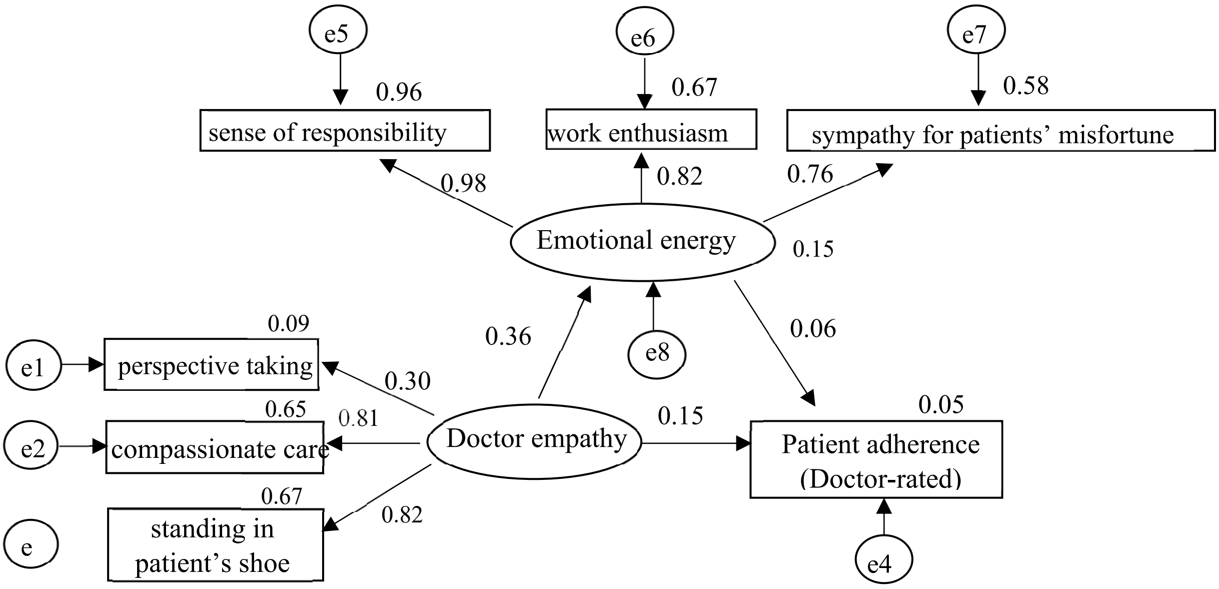

Figure S3. **Mediating role of emotional energy**

Note: The values shown in the figure are standardized estimates. Doctor's gender, age, marital status, and educational level were controlled during analysis. To maintain graphical clarity, the path analysis results are mainly showed the relationship between doctor empathy, emotional energy and doctor-rated patient adherence

Table S11. The regression coefficients of the mediating role of doctor treatment behavior

Pathways	Estimated values [a]	Estimated values [b]	S.E.	C.R.	*P*
Doctor empathy—Doctor treatment behavior	0.12	0.42	0.02	5.04	<0.001
Doctor empathy—Doctor-rated Patient adherence	0.07	0.16	0.03	2.37	0.018
Doctor treatment behavior—Patient adherence	0.08	0.05	0.09	0.90	0.367
Gender—Doctor treatment behavior	0.09	0.07	0.05	1.60	0.110
Age—Doctor treatment behavior	0.01	0.14	0.004	2.66	0.008
Marital status—Doctor treatment behavior	-0.12	-0.07	0.09	-1.30	0.193
Educational level—Doctor treatment behavior	-0.05	-0.03	0.07	-0.69	0.490
Gender—Doctor-rated Patient adherence	-0.16	-0.08	0.09	-1.76	0.079
Age—Doctor-rated Patient adherence	0.001	0.01	0.01	0.16	0.871
Marital status—Doctor-rated Patient adherence	0.17	0.06	0.16	1.08	0.282
Educational level—Doctor-rated Patient adherence	0.17	0.07	0.13	1.32	0.186

Note: C.R. (critical ratio) corresponds to the t-value, and S.E. indicates the standard error; a: estimates reflect unstandardized path coefficients, b: estimates reflect standardized path coefficients.

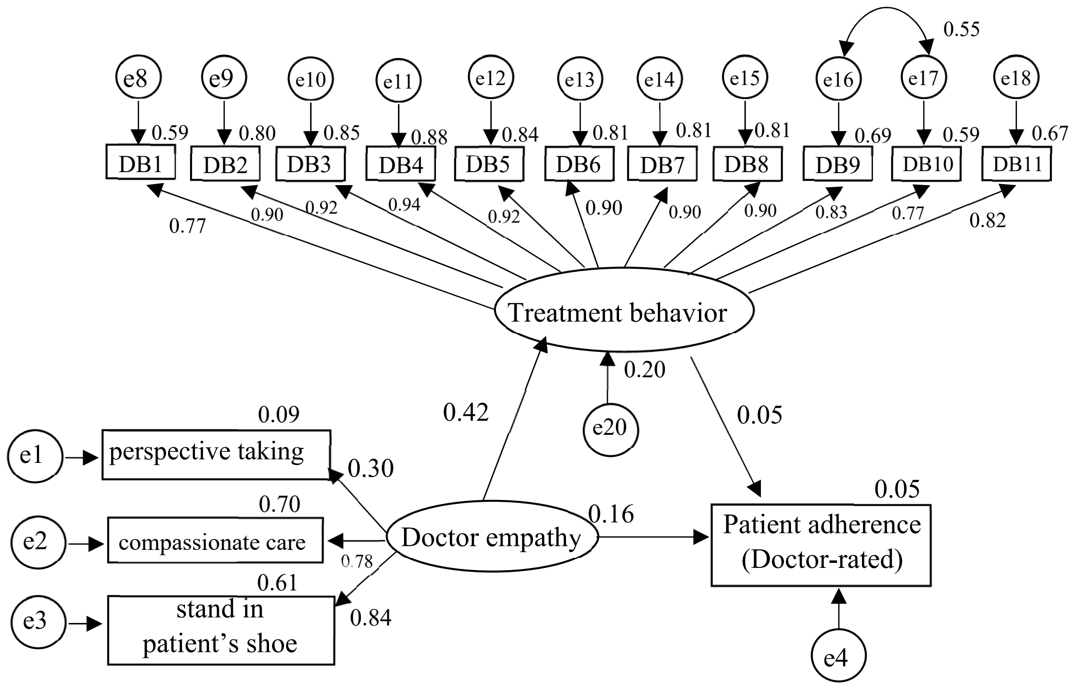

Figure S4. Mediating role of doctor treatment behavior

Note: The values shown in the figure are standardized estimates. Doctor's gender, age, marital status, and educational level were controlled during analysis. To maintain graphical clarity, the path analysis results are mainly showed the relationship between doctor empathy, doctor treatment behavior and doctor-rated patient adherence

Table S12. The regression coefficients of the chain mediating role of emotional energy and doctor treatment behavior

Pathways	Estimated values [a]	Estimated values [b]	S.E.	C.R.	*P*
Doctor empathy—Emotional energy	0.64	0.36	0.14	4.76	<0.001
Emotional energy—Doctor treatment behavior	0.12	0.78	0.01	17.26	<0.001
Doctor empathy—Doctor treatment behavior	0.04	0.15	0.01	3.52	<0.01
Doctor empathy— Doctor-rated Patient adherence	0.07	0.16	0.03	2.35	0.019
Emotional energy—Doctor-rated Patient adherence	0.01	0.04	0.02	0.44	0.657
Doctor treatment behavior—Doctor-rated Patient adherence	0.03	0.02	0.16	0.16	0.870
Gender—Emotional energy	0.26	0.03	0.36	0.72	0.475
Age—Emotional energy	0.07	0.16	0.03	2.93	0.003
Marital status—Emotional energy	-0.46	-0.04	0.61	-0.75	0.452
Educational level—Emotional energy	-0.19	-0.02	0.48	-0.40	0.690
Gender—Doctor-rated Patient adherence	0.05	0.04	0.03	1.55	0.122

Pathways	Estimated values [a]	Estimated values [b]	S.E.	C.R.	*P*
Age—Doctor-rated Patient adherence	0.001	0.02	0.002	0.49	0.623
Marital status—Doctor-rated Patient adherence	-0.07	-0.04	0.06	-1.24	0.215
Educational level—Doctor-rated Patient adherence	-0.02	-0.02	0.05	-0.50	0.619
Gender—Doctor-rated Patient adherence	-0.16	-0.08	0.09	-1.74	0.083
Age—Doctor-rated Patient adherence	0.001	0.01	0.01	0.13	0.895
Marital status—Patient adherence	0.17	0.06	0.16	1.07	0.287
Educational level—Doctor-rated Patient adherence	0.17	0.07	0.13	1.32	0.187

Note: C.R. (critical ratio) corresponds to the t-value, and S.E. indicates the standard error; a: estimates reflect unstandardized path coefficients, b: estimates reflect standardized path coefficients.

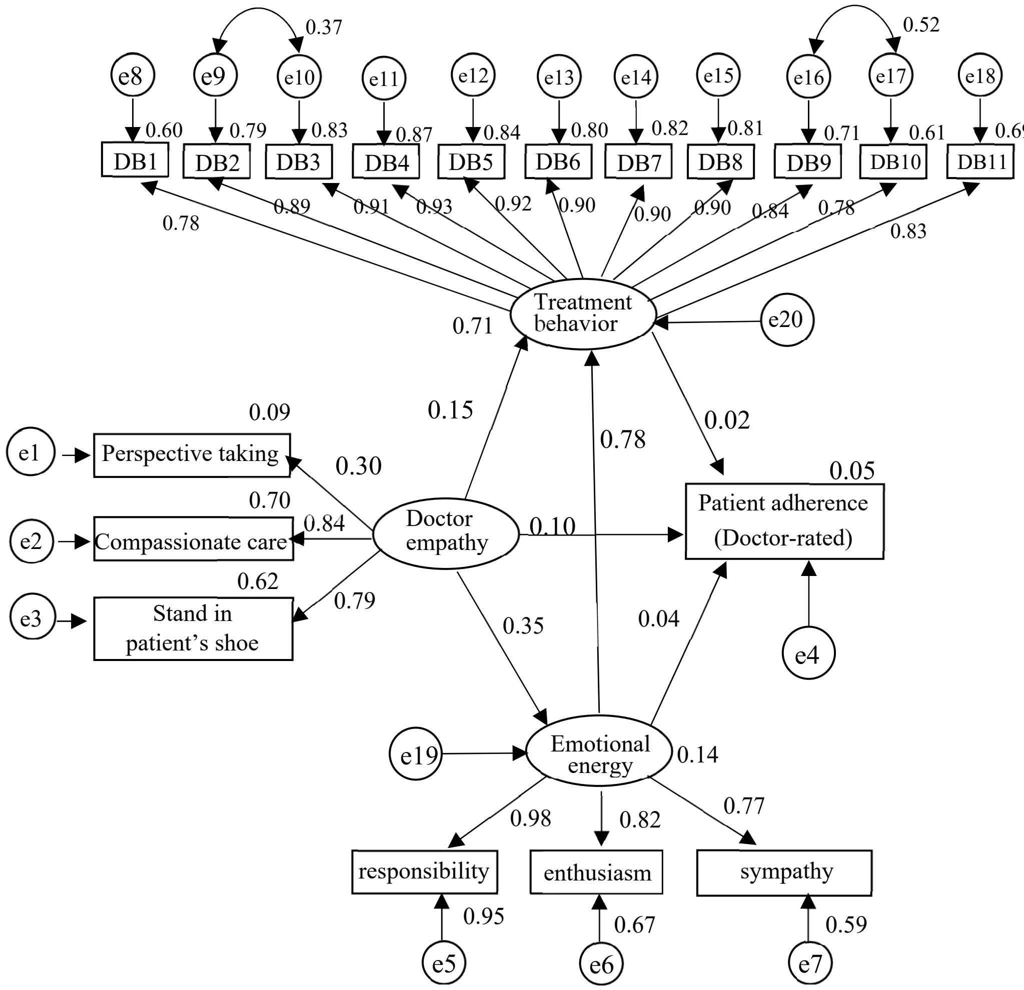

Figure S5. Mediating role of emotional energy and doctor treatment behavior

Note: The values shown in the figure are standardized estimates. Doctor's gender, age, marital status, and educational level were controlled during analysis. To maintain graphical clarity, the path analysis results are mainly showed the relationship between doctor empathy, doctor emotional energy, doctor treatment behavior and doctor-rated patient adherence

S5. Potential common method bias (CMB) analysis results of measurements

Harman's single-factor test was conducted via exploratory factor analysis (EFA) using SPSS 20.0. All scale items measuring core study variables—doctor empathy, doctor emotional energy, doctor treatment behaviors, and doctor-rated patient adherence—were simultaneously included in the analysis.

The Kaiser–Meyer–Olkin (KMO) statistic was 0.95, and Bartlett's test of sphericity yielded a χ^2 value of 15443.54 with $p < 0.001$, confirming the dataset was suitable for factor analysis. The unrotated solution extracted nine factors with eigenvalues greater than 1. The first unrotated common factor explained only 35.95% of the total variance, which fell below the critical threshold of 40% [1,2]. The cumulative explained variance across all retained factors reached 69.69%. These results indicated that common method bias within the physician questionnaire was within an acceptable range (see Table S13).

Table S13. Results of exploratory factor analysis for the doctor measurements

**Component**	**Initial Eigenvalues**			**Extraction Sums of Squared Loadings**			**Rotation Sums of Squared Loadings**		
	Total	% of Variance	Cumulative %	Total	% of Variance	Cumulative %	Total	% of Variance	Cumulative %
1	19.77	35.95	35.95	19.77	35.95	35.95	13.73	24.96	24.96
2	4.67	8.50	44.44	4.67	8.50	44.44	6.48	11.78	36.74
3	4.17	7.59	52.03	4.17	7.59	52.03	4.56	8.29	45.03
4	2.98	5.42	57.46	2.98	5.42	57.46	3.31	6.03	51.06
5	1.75	3.19	60.64	1.75	3.19	60.64	3.07	5.58	56.64
6	1.16	2.12	62.76	1.16	2.12	62.76	2.54	4.63	61.27
7	1.15	2.09	64.85	1.15	2.09	64.85	1.66	3.02	64.29
8	1.08	1.96	66.80	1.08	1.96	66.80	1.22	2.22	66.50
9	1.04	1.89	68.69	1.04	1.89	68.69	1.20	2.19	68.69
…	…	…		—	—	—	—	—	—
53	0.09	0.16	99.78	—	—	—	—	—	—
54	0.06	0.11	99.90	—	—	—	—	—	—

Component	Initial Eigenvalues			Extraction Sums of Squared Loadings			Rotation Sums of Squared Loadings		
	Total	% of Variance	Cumulative %	Total	% of Variance	Cumulative %	Total	% of Variance	Cumulative %
55	0.06	0.10	100.00	—	—	—	—	—	—

References of section 5:

1. Deng, W.; Li, X.; Chen, B.; Luo, K.; Zeng, X. Analysis on application of common methods bias test to psychological studies during recent five years in China. *J. Jiangxi Norm. Univ. (Nat. Sci.)* 2018, *42*, 447–453. https://10.16357/j.cnki.issn1000-5862.2018.05.02.(In Chinese)
2. Tan, D. Wen, Z. Statistical Approaches for Testing Common Method Bias: Problems and Suggestions.*Journal of Pyschological Science*,2020,*43*(01):215-223.https://10.16719/j.cnki.1671-6981.20200130.(in Chinese)
